# Long-Term Depression of Striatal DA Release Induced by mGluRs *via* Sustained Hyperactivity of Local Cholinergic Interneurons

**DOI:** 10.3389/fncel.2021.798464

**Published:** 2021-12-02

**Authors:** Nicola B. Mercuri, Mauro Federici, Francesca Romana Rizzo, Lorenzo Maugeri, Sebastian L. D’Addario, Rossella Ventura, Nicola Berretta

**Affiliations:** ^1^IRCCS Fondazione Santa Lucia, Laboratory of Experimental Neurology, Rome, Italy; ^2^Department of Systems Medicine, University of Tor Vergata, Rome, Italy; ^3^Department of Psychology and Center Daniel Bovet, Sapienza University, Rome, Italy; ^4^Behavioral Neuroscience PhD Programme, Sapienza University, Rome, Italy

**Keywords:** dopamine, LTD, striatum, cholinergic interneurons, metabotropic glutamate receptors, amperometry, multielectrode array recordings

## Abstract

The cellular mechanisms regulating dopamine (DA) release in the striatum have attracted much interest in recent years. By *in vitro* amperometric recordings in mouse striatal slices, we show that a brief (5 min) exposure to the metabotropic glutamate receptor agonist DHPG (50 μM) induces a profound depression of synaptic DA release, lasting over 1 h from DHPG washout. This long-term depression is sensitive to glycine, which preferentially inhibits local cholinergic interneurons, as well as to drugs acting on nicotinic acetylcholine receptors and to the pharmacological depletion of released acetylcholine. The same DHPG treatment induces a parallel long-lasting enhancement in the tonic firing of presumed striatal cholinergic interneurons, measured with multi-electrode array recordings. When DHPG is bilaterally infused *in vivo* in the mouse striatum, treated mice display an anxiety-like behavior. Our results demonstrate that metabotropic glutamate receptors stimulation gives rise to a prolonged depression of the striatal dopaminergic transmission, through a sustained enhancement of released acetylcholine, due to the parallel long-lasting potentiation of striatal cholinergic interneurons firing. This plastic interplay between dopamine, acetylcholine, and glutamate in the dorsal striatum may be involved in anxiety-like behavior typical of several neuropsychiatric disorders.

## Introduction

Dopamine (DA) is a catecholamine playing a crucial role in a variety of human and animal behaviors, spanning from control in the precision of movements to higher cognitive functions, including learning, memory, decision making, and seeking behavior associated to reward (Groenewegen, 2003; Nelson and Kreitzer, 2014; Schultz, 2016; Westbrook et al., 2021). The distinctive importance of this neurotransmitter becomes particularly evident when considering the relevance of neurological and psychiatric disorders whose symptoms are directly correlated with DA release disfunction, like Parkinson’s disease (PD), schizophrenia, addiction, and compulsive disorders (Lipski et al., 2011; Grace, 2016; Volkow et al., 2017; Wise and Robble, 2020). With regards to PD, in particular, depression and anxiety are among the most important non-motor signs of the disease and functional studies in humans have shown the DAergic system playing an important role in their manifestations (Weintraub et al., 2005; Thobois et al., 2017).

The major recipient of DA release in the forebrain is represented by the striatum, in its dorsal and ventral portions, by means of projections originating from the ventral midbrain, namely the substantia nigra pars compacta and the ventral tegmental area. Thus, it is mostly through the striatum that the DAergic transmission exerts its fundamental role in motor control and in cognitive behavior as well as in neuropsychiatric symptoms (Weintraub et al., 2005; Tritsch and Sabatini, 2012; Thobois et al., 2017).

With these premises, it is not surprising that much experimental effort has been put in recent years in trying to uncover all the subtle cellular and molecular mechanisms participating in the modulation of striatal DA release, through the convergence of intrinsic and extrinsic synaptic circuitries within the striatum (Zhang and Sulzer, 2004; Cachope and Cheer, 2014; Sulzer et al., 2016). For instance, DA controls its own release by means of D2 auto-receptors (Schmitz et al., 2003; Ford et al., 2010). With regards to excitatory amino-acids, glutamate may modulate DA release following activation of ionotropic or metabotropic glutamate receptors (mGluRs), through inputs arising from the thalamus or the cortex, or possibly from DAergic terminals co-releasing glutamate (Avshalumov et al., 2003, 2008; Zhang and Sulzer, 2003; Chuhma et al., 2004; Hnasko et al., 2010). Interestingly, mGluRs are involved in the pathogenesis of PD as their overactivity in the basal ganglia has been described in both PD animal models and PD patients (Rouse et al., 2000; Olanow, 2002). Last but not least, acetylcholine (ACh) released from local cholinergic interneurons (ChIs), exerts a delicate and essential role, mainly by stimulation of presynaptic nicotinic ACh receptors (nAChRs) on the DAergic terminals (Rice and Cragg, 2004; Cachope et al., 2012; Threlfell et al., 2012; Brimblecombe et al., 2018). Indeed, nAChRs facilitate DA release, however, a narrow window of extracellular ACh appears to be required, as depression of DA release can be achieved under higher ACh release, possibly due to nAChRs desensitization (Zhou et al., 2001; Wang et al., 2014).

As previously outlined, such modifications in DA release are believed to be important in terms of long-term modifications in the signal processing occurring at downstream synaptic circuitries. Noteworthily, most of the literature on long-term potentiation (LTP) or depression (LTD) has focused on fast glutamatergic or GABAergic synapses, whose induction or expression can be enabled or modulated by DA (Calabresi et al., 1996), as well as by several other neurochemical factors. However, apart from evidence of chronic alteration in the DAergic signal or of short-term dynamics of DA release (Condon et al., 2019), at present, it is not clear whether DAergic synapses in the striatum may be subject to acute synaptic plasticity processes, leading to sustained, long-term modifications in their efficacy, in a similar manner to glutamatergic or GABAergic synapses.

Here, we present evidence that DA release may undergo a sustained depression (DA-LTD), in response to 5 min exposure to the group I mGluR agonist DHPG, in a similar manner to the chemically-induced LTD reported at glutamatergic synapses (Fitzjohn et al., 2001; Sergeeva et al., 2007). However, differently from the latter, DA-LTD is not expressed by long-term modifications on the DA terminal proper, but by an indirect action linked to a sustained increase in the release of ACh, caused by a long-lasting increase in the excitability of the local ChIs.

## Methods

### Slice Preparation

Cortico-striatal slices were obtained from C57BL/6J mice (2–3 months) as described (Geracitano et al., 2003). We found no evidence of dissimilarity in the results obtained *in vitro* according to sex, thus, both males and females have been used in electrophysiological and amperometric recordings, while only male mice for the behavioral tests. The use of the animal was approved by the animal committee of the IRCCS- Fondazione Santa Lucia and followed the current Italian regulation of the Minister of Health. Briefly, coronal slices (250–300 μm) including neocortex and striatum were cut in artificial cerebrospinal fluid (aCSF) from tissue blocks by use of a vibratome (Leica VT1200-S, Leica Biosystems, Germany) and left to recover in aCSF for at least 1 h. A single slice was transferred to a recording chamber and submerged in a continuously flowing aCSF (2–3 ml/min for amperometric recordings and 5–6 ml/min for MEA recordings) composed of (in mM): 126 NaCl, 2.5 KCl, 1.2 NaH_2_PO_4_, 1.2 MgCl_2_, 2.4 CaCl_2_, 11 glucose, 25 NaHCO_3_ (T° 32–34°C, saturated with 95% O_2_ and 5% CO_2_).

### Amperometric Recordings

Constant current amperometric detection of DA was performed using a carbon fiber electrode (diameter 30 μm, length 100 μm, World Precision Instruments) placed to a depth of 50–150 μm into the dorsolateral striatum, as previously described (Federici et al., 2013). The imposed voltage (MicroC potentiostat, World Precision Instruments) between the carbon fiber electrode and an Ag/AgCl pellet was 550 mV. Evoked DA release was measured under electrical stimulation with a single rectangular electrical pulse through a bipolar Ni/Cr extracellular stimulating electrode, delivered by a DS3 Stimulator (Digitimer). Following a protocol of increasing stimulation intensity (20–1,000 μA, 40 μs), a plateau of DA release was reached. A test stimulation intensity was then selected, attaining 75% of the maximal amplitude and delivered every 5 min. Signals were digitized with Digidata 1440A coupled to a computer running pClamp 10 (both from Molecular Devices). Electrode calibration was performed at the end of each experiment by bath-perfused DA (0.3–10 μM), giving a sensitivity of 10–12.5 nM/pA.

### Multielectrode Array Recordings

The cortico-striatal slice was placed over an 8 × 8 array of planar electrodes, each 50 × 50 μm in size, with an interpolar distance of 150 μm (MED-P5155, Alpha MED Sciences), so that most of the dorsolateral portion of the striatum was covered by underlying electrodes. The slice was kept submerged in continuously flowing aCSF by a platinum ring covered with nylon mesh (see Figure 4A). Voltage signals were recorded with the MED64 System (Alpha MED Sciences), digitized at 20 kHz, and filtered at 0.1–1 Hz with a 6071E Data Acquisition Card (National Instruments), using Mobius software (Alpha MED Sciences). The channels presenting fast transients (see Figure 4B), indicative of tonic spontaneous action potentials, were selected and stored for off-line analysis with Spike2 6.0 software (Cambridge Electronic Design Ltd, Cambridge, UK). The spikes were acquired by means of an amplitude threshold adjusted by visual inspection. When more than one neuron was detected by the same channel, spike-sorting discrimination was achieved according to the shape of the spike waveforms using the same software, with a normal mixtures algorithm on independent clusters obtained from principal component data (Berretta et al., 2010).

**Figure 1:**
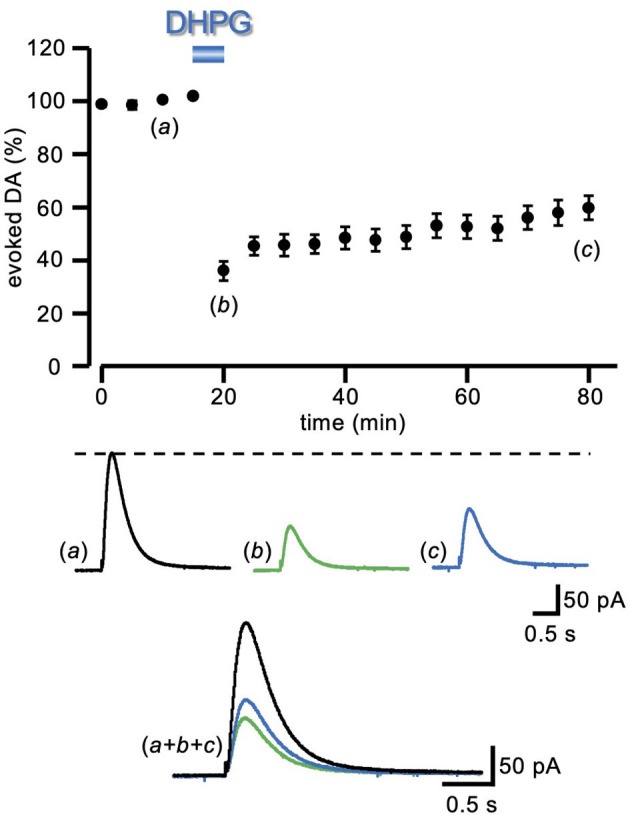
LTD of evoked DA induced by DHPG. Running plots of the relative (%) mean amplitude (± s.e.m.) of evoked DA release, obtained with amperometric recordings in striatal slices, with a local electrical stimulation delivered at 5 min intervals (*n* = 23 slices from nine animals). Bath perfusion with DHPG (50 μM) for 5 min caused a pronounced depression of the DA signal, which remained depressed after 60 min of drug washout. At the bottom, representative traces of the DA signal are shown at the time indicated by the letters in the plot. LTD, long-term depression; DA, dopamine; DHPG, (S)-3, 5-dihydroxyphenylglycine.

**Figure 2:**
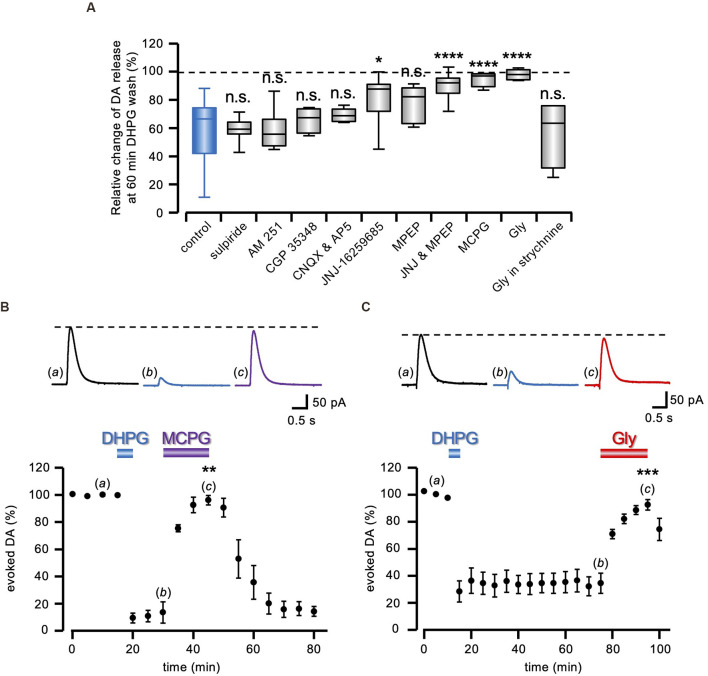
Pharmacology of DA-LTD. **(A)** Box and whiskers plot of the change in evoked DA release, 60 min after DHPG (50 μM) washout, relative to the DA signal preceding the DHPG treatment, in the presence of 1 μM sulpiride (*n* = 15 slices from six animals), 5 μM AM 251 (*n* = 7 slices from three animals), 200 μM CGP 35348 (*n* = 4 slices from two animals), 10 μM CNQX (*n* = 6 slices from three animals), 500 nM JNJ-16259685 (*n* = 9 slices from four animals), 300 nM MPEP (*n* = 9 slices from four animals), co-perfusion of JNJ and MPEP (*n* = 11 slices from five animals), 1 mM MCPG (*n* = 4 slices from two animals), 1 mM Gly (*n* = 8 slices from four animals) and Gly in 1 μM strychnine (*n* = 4 slices from two animals). All statistical comparisons are made against the control condition (blue box), i.e., DHPG treatment with no additional drug illustrated in Figure 1 (n.s. *P* > 0.05, *P < 0.05, ****P < 0.0001, 1-way ANOVA followed by Bonferroni’s test). **(B,C)** Running plots of the mean relative amplitude (± s.e.m.) of evoked DA release, obtained with amperometric recordings at 5 min stimulation interval. On top, representative traces of the DA signal are shown at the time indicated by the letters in the plots. DA-LTD expression after DHPG (50 μM) washout was reverted in **(B)** by 1 mM MCPG (*n* = 4 slices from two animals) and in **(C)** by 1 mM Gly (*n* = 5 slices from two animals). **P < 0.01, ***P < 0.001, Student’s t-test for paired data, between evoked DA amplitude during and immediately before drug perfusion.

**Figure 3:**
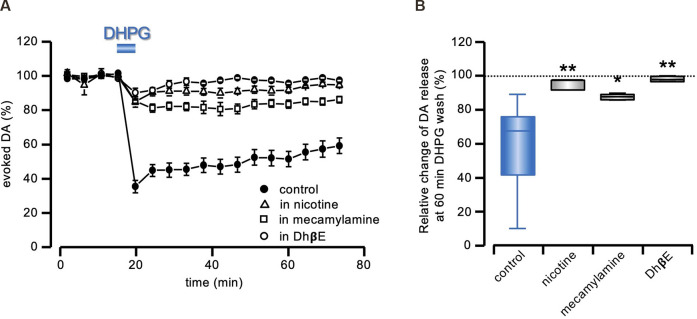
DA-LTD prevented by nAChRs stimulation or inhibition. **(A)** Running plots of the mean relative amplitude (± s.e.m.) of evoked DA release, obtained with amperometric recordings at 5 min stimulation interval, in response to a 5 min exposure to DHPG (50 μM), in control conditions, or in the continuous presence of 100 nM nicotine (*n* = 5 slices from three animals), 500 nM mecamylamine (*n* = 4 slices from two animals) or 1 μM DhβE (*n* = 4 slices from two animals). The control plot is the same as illustrated in Figure 1. **(B)** Box and whiskers plot of the change in evoked DA release, 60 min after DHPG (50 μM) washout, relative to the DA signal preceding the DHPG treatment, in the presence of the drugs indicated on the abscissa. All statistical comparisons are made against the same control as in Figure 2B (blue box), i.e., DHPG treatment with no additional drug (*P < 0.05; **P < 0.01, 1-way ANOVA followed by Bonferroni’s test).

**Figure 4:**
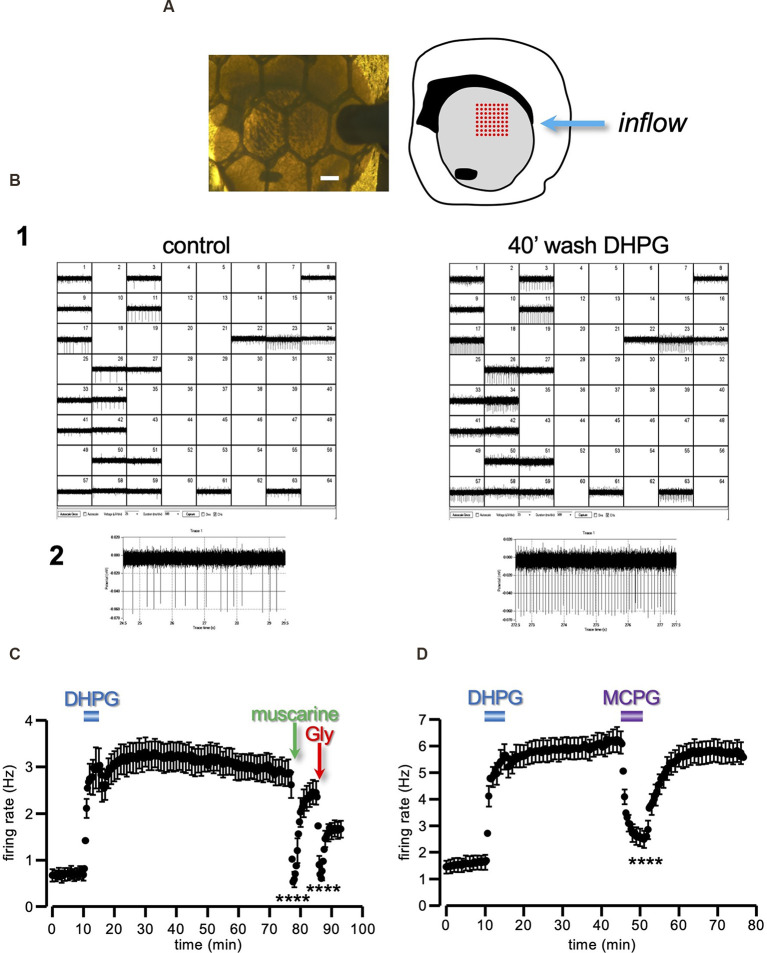
Long-lasting enhancement of ChIs firing induced by DHPG. **(A)** Photograph of a coronal striatal slice covered by a nylon mesh and continuously perfused by aCSF, with its schematic drawing on the right. The slice was placed in an MEA recording chamber over an 8 × 8 array of planar electrodes (red dots in the drawing representation), in such a way as to cover the dorsal portion of the striatal area (grey area of the drawing representation). Scale bar 0.5 mm. **(B1)** Out of the total 64 channels, only those containing repetitive fast deflections, indicative of spontaneous action potentials, were selected for analysis (22 channels in this example). Note the generalized increase in the spontaneous firing recorded 40 min after DHPG (50 μM) washout, compared to control. One of the 22 active channels is highlighted in **(B2)**. **(C,D)** Running plots of the firing rate (mean ± s.e.m. binned every 20 s) of spontaneously active neurons in striatal slices. Note the long-lasting increase in the firing rate caused by a 5 min perfusion with DHPG (50 μM). **(C)** The spontaneous firing was sensitive to muscarine (30 μM) and glycine (Gly; 1 mM), as expected from spikes originated by the ChIs population (*n* = 39, from five slices and three animals). **(D)** The sustained potentiation of the firing rate expressed at DHPG washout was reversibly reverted by 1 mM MCPG (*n* = 33, from two slices and two animals). ****P < 0.0001, Student’s t-test for paired data, between firing rate during and immediately before drug prefusion. CHIs, cholinergic interneurons; aCSF, artificial cerebrospinal fluid.

### Stereotaxic Injection

Adult male mice (10–15-week-old) were anesthetized with Zoletil 100 (tiletamine HCl 50 mg/ml + zolazepam HCl 50 mg/ml; from Virbac, Italy) and Rompun 20 (xylazine 20 mg/ml; from Bayer S. p. A, Italy) dissolved in saline solution (4.1 mg/ml and 1.6 mg/ml, respectively) prior to injection (7.3 ml/kg). Mice were then positioned in a stereotaxic frame (David Kopf Instruments, CA, USA) equipped with a mouse adapter. For behavioral experiments, mice were chronically implanted bilaterally with a 26-gauge guide cannula positioned 1 mm from the dorsolateral striatum, aiming at the same striatal area investigated *in vitro*, using the following coordinates (from brain surface): AP = +1 mm, ML = ±1.5 mm, DV: −3.25 mm (Robins et al., 2018). After 5–7 days of post-operative recovery, DHPG (50 μM in NaCl 0.9%) or vehicle (Veh, NaCl 0.9%) treated animals were bilaterally injected through an injector cannula in a total volume 0.5 μl/side at a continuous rate of 0.15 μl/min under the control of a micro-infusion pump. The injector cannula was removed 3 min after the end of infusion to prevent backflow. Mice were returned to their home cage and tested after 10 min.

### Elevated Plus Maze (EPM)

Mice were individually tested in a 5 min session in an apparatus elevated of 38.5 cm above the floor and consisting of two open arms (27 × 5 cm) and two closed arms (27 × 5 × 15 cm) connected by a central platform (5 × 5 cm) representing the starting point (Di Segni et al., 2019). The percentage of entries in the open arms (closed entries/open + closed × 100), the percentage of time spent in the open arms (time in open/open + closed × 100), and distance moved (cm) were collected and analyzed by the “EthoVision” (Noldus, The Netherlands) fully automated video tracking system.

### Open Field (OF)

Mice were introduced in the central sector and free to explore the apparatus consisting in a circular open field, 60 cm in diameter, and 20 cm in height for 5 min (Di Segni et al., 2019). The percentage of time spent in center and distance moved (cm), was video recorded and analyzed by the “EthoVision” (Noldus, The Netherlands) fully automated video tracking system.

### Data Analysis

Quantitative data are expressed as mean (± s.e.m.). Student’s *t*-test for paired or unpaired observations was used to compare the means of two data points in the same experimental protocol or from two separate protocols, respectively. 1-way ANOVA, followed by Bonferroni’s multiple comparison test, was used when multiple comparisons were made against a single control group (Origin pro 2019, Northampton, MA, USA). The difference was considered significant at levels of *P* < 0.05.

### Drugs

All drugs were dissolved to the desired final concentration in aCSF. These included: *N*-(Piperidin-1-yl)-5-(4-iodophenyl)-1-(2,4-dichlorophenyl)-4-methyl-1*H*-pyrazole-3-carboxamide (AM 251), D(-)-2-amino-5-phosphonopentanoic acid (AP5), 3-aminopropyl diethoxymethyl phosphinic acid (CGP 35348), 6-cyano-7-nitroquinoxaline-2,3-dione (CNQX), dihydro-β-erythroidine hydrobromide (DhBE), (S)-3,5-dihydroxyphenylglycine (DHPG), mecamylamine hydrochloride, S-(-)-methyl-4-carboxyphenylglycine (MCPG), 2-methyl-6-(phenylethynyl)-pyridine (MPEP), 3,4-dihydro-2*H*-pyrano[2,3-*b*]quinolin-7-yl-(*cis*-4-methoxycyclohexyl)-methanone (JNJ-16259685) from Tocris Bio-Techne ltd (Milan, Italy); glycine, hemicholinium-3, muscarine hydrochloride, neostigmine bromide, nicotine bitartrate dihydrate, strychnine hydrochloride, (±) sulpiride and vesamicol hydrochloride from Merk Life Science S.r.l. (Milan, Italy).

## Results

### Long-Term Depression of DA Release Induced by DHPG

Electrical stimulation of striatal slices by a single pulse caused the synaptic release of DA that was recorded as an amperometric wave. Following a baseline period of at least 20 min, in order to assess the stability of evoked DA release, DHPG (50 μM) was added into the perfusing medium for 5 min, and changes relative to baseline were evaluated. In the presence of DHPG (50 μM), a sharp reduction of evoked DA release was observed (35.98 ± 3.60% of control), that only partially recovered at DHPG washout, attaining (59.72 ± 4.59% of control, *n* = 23 slices from nine animals) after 60 min (Figure 1), giving rise to a DHPG-induced long-term depression of the DAergic transmission (DA-LTD).

### Pharmacology of DHPG-Induced DA-LTD

As shown in the summary box and whiskers plot of Figure 2A, we compared DA-LTD 60 min after DHPG (50 μM) washout in control conditions and co-applied under a battery of pharmacological treatments (*F*_10,89_= 9.80; *P* < 0.0001, 1-way ANOVA). According to *post hoc* analysis with Bonferroni’s test, we found that DA-LTD was not significantly different from that induced with no added drugs, in the presence of 1 μM sulpiride (60.09 ± 1.85% of control; *t* = 0.07, *P* > 0.95, *n* = 15 slices from six animals), 5 μM AM 251 (59.36 ± 5.36% of control; *t* = 0.06, *P* > 0.95, *n* = 7 slices from three animals) and 200 μM CGP 35348 (66.62 ± 4.55% of control; *t* = 0.86, *P* > 0.95, *n* = 4 slices from two animals), ruling out a role of D2, CB1, and GABAB receptors, respectively. Ionotropic glutamate receptors were similarly excluded, as DA-LTD was still present in the presence of 50 μM AP5 and 10 μM CNQX (70.12 ± 1.89% of control; *t* = 1.54, *P* > 0.95, *n* = 6 slices from three animals). Conversely, the broad-spectrum group I mGluR antagonist MCPG (1 mM) prevented DHPG-elicited DA-LTD (96.31 ± 2.79% of control; *t* = 4.58, *P* < 0.0001, *n* = 4 slices from two animals), and a reduced DA-LTD expression was observed either under the mGluR1 antagonist 500 nM JNJ-16259685 (82.34 ± 5.54% of control; *t* = 3.94, *P* < 0.05, *n* = 9 slices from four animals) or the mGluR5 antagonist 300 nM MPEP, although still not significantly different from control (76.96 ± 4.31% of control; *t* = 2.98, *P* = 0.21, *n* = 9 slices from four animals). Conversely, co-perfusion of both mGluRs agonists prevented DA-LTD (91.27 ± 2.79% of control; *t* = 5.84, *P* < 0.0001, *n* = 11 slices from five animals). Similarly, the inhibitory amino acid glycine (Gly; 1 mM) prevented DA-LTD induced by DHPG (99.36 ± 1.27% of control; *t* = 6.55, *P* < 0.0001, *n* = 8 slices from four animals), in a 1 μM strychnine-sensitive manner (57.61 ± 12.28% of control; *t* = 0.26, *P* > 0.95, *n* = 4 slices from two animals).

Interestingly, in agreement with what was observed for DHPG-induced LTD of the glutamatergic transmission (Lodge et al., 2013), MCPG (1 mM) not only prevented DA-LTD induction, but it also reverted its expression, as it restored DA transmission to control level, once previously induced (96.06 ± 3.41% of control, *n* = 4 slices from two animals; *t* = 7.59, *P* < 0.01 Student’s t-test; Figure 2B). The same effect was observed under 1 mm Gly during a previously established DA-LTD (92.56 ± 3.89% of control, *n* = 5 slices from two animals; *t* = 11.55, *P* < 0.001 Student’s t-test; Figure 2C), indicating that DA-LTD requires the sustained participation of cellular mechanisms sensitive to Gly receptor-mediated inhibition.

### Role of nAChRs in DHPG-Mediated DA-LTD

According to published literature (Darstein et al., 2000; Waldvogel et al., 2007), Gly receptors in the striatum are mainly expressed by the local population of cholinergic interneurons (ChIs). In view of the notorious subtle interplay between DA and ACh release in the striatum, involving nAChRs stimulation on the DAergic terminals (Zhou et al., 2001; Wang et al., 2014; Brimblecombe et al., 2018), we investigated whether the sustained reduction of DA release by mGluRs stimulation could be a secondary effect to modifications in the local cholinergic signal.

In agreement with previous reports (Zhou et al., 2001; Wang et al., 2014; Rizzo et al., 2018), bath perfusion with nicotine (100 nM), reduced evoked DA release. We compensated this depression with a 20–40% increase in afferent stimulation intensity. In these conditions, under the continuous presence of nicotine (100 nm), the DA signal did not undergo long-lasting modifications after 5 min exposure in 50 μM DHPG (95.59 ± 1.27% of control; *F*_3,32_= 9.91; *P* < 0.0001, 1-way ANOVA and *t* = 3.98, *P* < 0.01, Bonferroni’s test, *n* = 5 slices from three animals; Figure 3).

We then tested the sensitivity of DA-LTD to the broad spectrum nAChR antagonist mecamylamine (500 nM) and to the α4β2 subunit-selective nAChR antagonist DhβE (1 μM). As expected (Zhou et al., 2001; Wang et al., 2014; Rizzo et al., 2018), both antagonists reduced evoked DA release, which was compensated as above. As shown in Figure 3, no DA-LTD was induced by 50 μM DHPG under the continuous presence of either 500 nM mecamylamine (87.61 ± 1.00% of control; *F*_3,32_= 9.91; *P* < 0.0001, 1-way ANOVA and *t* = 2.82, *P* < 0.05, Bonferroni’s test, *n* = 4 slices from two animals) or 1 μM DhβE (98.05 ± 0.76% of control; *F*_3, 32_= 9.91; *P* < 0.0001, 1-way ANOVA and *t* = 3.87, *P* < 0.01, Bonferroni’s test, *n* = 4 slices from two animals).

### Sustained Striatal ChIs Firing Rate Potentiation by mGluRs Stimulation

Assuming a sustained upturn in the level of ambient ACh in response to DHPG, as a cellular substrate of the sustained DA releases depression, we predicted that the same 5 min DHPG treatment should give rise to parallel and congruent modifications in the excitability of the local ChIs population. With this aim, we conducted experiments using a multielectrode array (MEA) device on the same cortico-striatal slice (Figures 4A,B). This technique is highly sensitive and capable to detect the spontaneous firing of a large number of locally active neurons (Berretta et al., 2010). The large majority of striatal neurons belong to the projecting medium spiny population, however, in a slice preparation, these are typically silent due to the lack of extrinsic excitatory inputs (Nisenbaum and Wilson, 1995). Therefore, most of the spontaneous spikes detected with MEA are expected to arise from local tonically active neurons belonging to the ChIs population, although the participation of GABAergic interneurons cannot be excluded.

The firing rate of the striatal tonically active neurons recorded had a baseline firing of 0.69 ± 0.13 Hz (*n* = 39, from five slices and three animals), consistent with the low spontaneous rate of the ChIs reported in the literature (Bennett and Wilson, 1999; Bennett et al., 2000). When the slices were exposed to DHPG (50 μM) for 5 min, a sharp increase in the firing rate to 2.96 ± 0.45 Hz was observed. More interestingly, the firing rate did not recover to the baseline but remained potentiated relative to control for over 60 min from DHPG washout (2.91 ± 0.29 Hz; Figure 4C).

In order to confirm that we were recording from ChIs, we applied muscarine (30 μM) and glycine (Gly; 1 mM). Indeed, M2/M4 muscarinic ACh receptors (mAChRs) and GlyRs are densely concentrated at ChI postsynaptic and non-synaptic regions, leading to membrane potential hyperpolarization (Weiner et al., 1990; Bernard et al., 1992; Darstein et al., 2000; Sergeeva and Haas, 2001; Waldvogel et al., 2007; Bonsi et al., 2008; Zhao et al., 2016). As expected, both muscarine (*t* = 9.99, *P* < 0.0001 Student’s t-test) and Gly (*t* = 9.54, *P* < 0.0001 Student’s t-test) produced an abrupt and significant reduction of the spontaneous firing (Figure 4C).

These results show that a brief exposure to DHPG induces a sustained increase of the basal firing rate of presumed striatal ChIs, with a time-course reflecting the previously observed depression of the DA response induced by DHPG.

In keeping with the parallel expression of DA-LTD and sustained increase of tonically active neurons firing rate, the broad-spectrum group I mGluR antagonist MCPG (1 mM) reversibly reverted the sustained potentiation of the firing rate induced by a previous 5 min treatment with DHPG (Figure 4D; *n* = 33, from two slices and two animals; *t* = 8.72, *P* < 0.0001 Student’s t-test).

### DA-LTD Prevented by ACh Depletion

According to the hypothesis that the long-term effect induced by group I mGluRs stimulation on the striatal DA transmission is caused by a sustained increase in ACh release, due to the sustained increase of ChIs firing, we predicted that, in the condition of ACh depletion, DHPG effect on evoked DA release should be prevented. Thus, we preincubated the cortico-striatal slices with the vesicular ACh transporter antagonist vesamicol (Rícný and Collier, 1986) and the choline reuptake inhibitor hemicholinium-3 (Parikh and Sarter, 2006).

**Figure 5:**
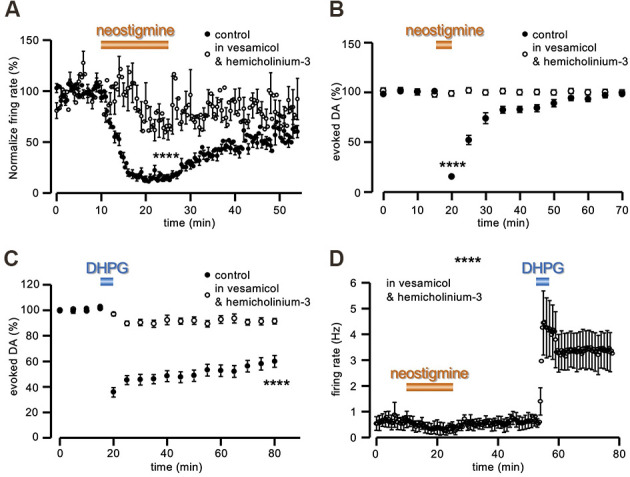
ACh depletion prevents DA-LTD. **(A)** Running plots of the firing rate (mean ± s.e.m. binned every 20 s) of spontaneously active neurons in striatal slices, recorded with MEA. While in control conditions neostigmine (1 μM) inhibited ChIs firing (*n* = 70, from four slices and three animals), this effect was reduced in slices preincubated for at least 2 h and continuously perfused in 10 μM vesamicol and 10 μM hemicholinium-3 (*n* = 52, from six slices and three animals). **(B,C)** Running plot of the relative mean amplitude (± s.e.m.) of evoked DA release, obtained with amperometric recordings at 5 min intervals. **(B)** Neostigmine (1 μM) reversibly inhibited evoked DA release in control conditions (*n* = 6 slices from three animals), while no change was observed in slices preincubated for at least 2 h and continuously perfused in 10 μM vesamicol and 10 μM hemicholinium-3 (*n* = 6 slices from three animals). **(C)** No DA-LTD was observed in slices preincubated for at least 2 h and continuously perfused in 10 μM vesamicol and 10 μM hemicholinium-3, following 5 min perfusion with 50 μM DHPG (*n* = 12 slices from six animals). The control plot is the same as illustrated in Figure 1. **(D)** Under the same conditions of vesamicol (10 μM) and hemicholinium-3 (10 μM), neostigmine (30 μM) produced a slight firing inhibition of presumed ChIs recorded with MEA (as in panel **A**), while the sustained increase of the firing rate in response to 5 min perfusion with DHPG (50 μM) was still evident (*n* = 6, from one slice and one animal). ****P < 0.0001, Student’s t-test for unpaired data.

The functional efficacy of this treatment was first tested with MEA recordings. In control conditions, the firing rate of the tonically active neurons recorded was inhibited to 14.71 ± 2.73% of control after rising tonic ACh with the cholinesterase inhibitor neostigmine (1 μM; *n* = 70, from four slices and three animals; Figure 5A), in agreement with the identity of the recorded neurons as presumed striatal ChIs (Calabresi et al., 1998; Scarduzio et al., 2017). Conversely, in slices preincubated for at least 2 h and continuously perfused in vesamicol (10 μM) and hemicholinium-3 (10 μM), the same exposure to neostigmine resulted in a significantly smaller firing inhibition (to 71.57 ± 12.09% of control; *n* = 52, from six slices and three animals; *t* = 5.22, *P* < 0.0001 Student’s t-test; Figure 5A), as expected under reduced ACh outflow. Likewise, in amperometric recordings, perfusion in neostigmine (1 μM) produced a profound inhibition of the evoked DA signal (15.37 ± 2.05% of control, *n* = 6 slices from three animals; Figure 5B), confirming that increased levels of extracellular ACh inhibit synaptic DA release. Conversely, in slices preincubated and continuously perfused in 10 μM vesamicol and 10 μM hemicholinium-3, the effect of neostigmine (1 μM) was significantly reduced (97.49 ± 2.95% of control, *n* = 6 slices from three animals; *t* = 29.71, *P* < 0.0001 Student’s t-test; Figure 5B). Notably, in striking contrast to DHPG, a complete recovery of the DA signal was observed at neostigmine washout, in control conditions, in spite of the profound inhibition caused by the drug.

In these experimental conditions of vesamicol (10 μM) and hemicholinium-3 (10 μM) pretreatment, 5 min perfusion in DHPG (50 μM) produced a significantly smaller depression of evoked DA release, compared to untreated slices (91.85 ± 2.85% of control at 60 min DHPG washout, *n* = 12 slices from six animals; *t* = 4.79, *P* < 0.0001 Student’s t-test; Figure 5C). Conversely, this pharmacological pre-treatment did not prevent the sustained increase in the firing rate of striatal tonically active neurons by DHPG (*n* = 6, from 1 slice and one animal; Figure 5D).

Thus, we were able to uncouple the increase of presumed ChIs firing from its functional outcome in terms of ACh release, and in so doing, DA-LTD was no longer present.

### Striatal DHPG Infusion Affects Anxiety-Like Behavior

**Figure 6:**
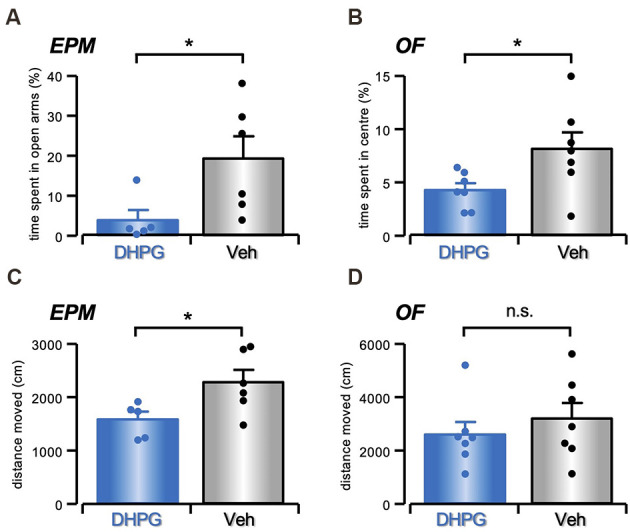
Anxiety-like behavior in DHPG-infused mice. **(A)** Histogram with single data points showing in % (mean ± s.e.m.) the time spent in the open arms of Elevated Plus Maze by DHPG (50 μM in NaCl 0.9%, *n* = 5) or Veh (NaCl 0.9%, *n* = 6) treated mice (*P < 0.05, Student’s t-test). **(B)** Histogram with single data points showing in % (mean ± s.e.m.) the time spent in the center of Open Field by DHPG- (*n* = 7) and Veh-treated (*n* = 7) mice (*P < 0.05, Student’s t-test). **(C)** Histogram with single data points showing in % (mean ± s.e.m.) the locomotor activity measured as total distance traveled (cm) over the arms in Elevated Plus Maze in DHPG- (*n* = 5) and Veh-treated (*n* = 6) mice (*P < 0.05, Student’s t-test). **(D)** Histogram with single data points showing in % (mean ± s.e.m.) the locomotor activity measured as total distance traveled (cm) over the Open Field in DHPG- (*n* = 7) and Veh-treated (*n* = 7) mice (n.s. *P* > 0.05, Student’s t-test).

With the aim to uncover behavioral modifications reflecting the results obtained *in vitro*, we performed *in vivo* bilateral DHPG infusions into the mouse striatum. This treatment produced an increase in anxiety-like behavior, as evident in both Elevated Plus Maze (EPM) and Open Field (OF). Analysis of the time spent in the open arms of EPM shows a difference between DHPG (50 μM in NaCl 0.9%; *n* = 5) and vehicle- (Veh, NaCl 0.9%; *n* = 6) treated mice with decreased time spent in the open arms shown by DHPG-treated in comparison with Veh-treated mice (*t* = 2.32, *P* < 0.05, Student’s t-test; Figure 6A). No significant difference was evident for the percentage of entries in open arms between DHPG-treated and Veh-treated mice (*t* = 1.37, P = n.s., Student’s t-test). Moreover, analysis of time spent in the central area of OF test shows a significant difference between DHPG- (*n* = 7) and Veh-treated mice (*n* = 7), with DHPG-treated mice spending less time in the central area compared to Veh-treated mice (*t* = 2.32, *P* < 0.05, Student’s t-test; Figure 6B).

No significant effect on locomotor activity was evident between DHPG- (*n* = 7) and Veh-treated mice (*n* = 7) in the OF test (*t* = 0.79, P = n.s., Student’s t-test; Figure 6D). However, the distance moved in EPM showed a significant difference between groups (*t* = 2.4, *P* < 0.05, Student’s t-test; Figure 6C), with reduced distanced moved observed by DHPG-treated (*n* = 5) in comparison with Veh-treated mice (*n* = 6).

## Discussion

In the present work, we provide evidence that the striatal DAergic transmission, measured with amperometric recordings of evoked DA release, is subject to a prolonged depression in its efficacy, in response to chemical stimulation of group I mGluRs by DHPG. This form of long-term synaptic depression (DA-LTD) is analogous, in its modality of induction and its temporal profile, to LTD of the glutamatergic transmission reported in cortical and striatal synapses (Bortolotto et al., 1999; Fitzjohn et al., 2001; Sergeeva et al., 2007).

A sustained depression of somato-dendritic DA release has previously been described in DAergic neurons of the ventral midbrain, evaluated from isolated D2 receptor-mediated postsynaptic currents, in response to a low-frequency stimulation protocol (Beckstead and Williams, 2007). Our report adds to these previous data, confirming the DAergic transmission as a leading actor, rather than just a supporter of long-term plasticity processes occurring in its targeted regions. The mechanism, though, underlying DA-LTD of our report seems to be different from that previously described in the SNpc. In fact, the latter was independent of activation of ionotropic or metabotropic glutamate receptors, as well as nAChRs (Beckstead and Williams, 2007).

### Mechanisms of DHPG-Induced DA-LTD

Striatal DAergic axons express mGluRs (Paquet and Smith, 2003), whose stimulation by glutamate spillover may cause a short-lasting depression of DA release (Zhang and Sulzer, 2003). These premises argue in favor of a similar mechanism to explain our present form of DA-LTD, induced by the group I mGluR agonist DHPG. Conversely, our results indicate an indirect mechanism, involving the local population of ACh-releasing interneurons. Indeed, we found that DA-LTD expression was reverted by glycine, an inhibitory amino acid whose receptors in the striatum are mainly expressed by the ChIs population (Darstein et al., 2000; Waldvogel et al., 2007). Moreover, not only was DA-LTD prevented by nAChR antagonists like mecamylamine and DhβE, but under nicotine itself was the DA response insensitive to DHPG, possibly through a mechanism of desensitization, while Zhang and Sulzer (2003) report an additional inhibition by DHPG under nAChRs block. Notably, we could still observe a small depression of the DA response in nicotine, under nAChRs block or acetylcholine depletion (see Figures 3 and 5A), thus, the most conceivable explanation to this apparent discrepancy is the coexistence of a direct mechanism of DA depression through presynaptic mGluRs on DAergic terminals and an indirect one involving ChIs, with a much stronger effect from the latter.

ChIs role in DA-LTD is further supported by the observation that the same protocol of 5 min DHPG bath exposure leads to a persistent increase in the excitability of striatal tonically active neurons, measured as an increase in their tonic firing rate, by means of MEA recordings. Although this technique does not allow their unequivocal identification as ChIs, their basal firing rate and their sensitivity to firing inhibition by muscarine (Bonsi et al., 2008; Zhao et al., 2016) and neostigmine (Scarduzio et al., 2017), strongly support this assumption. Both DA-LTD and potentiation of these presumed ChIs firing persisted over 1 h from DHPG washout, were sensitive to Gly receptors stimulation and their expression was reversibly inhibited by perfusion with the group I mGluRs antagonist MCPG.

The causal link between the sustained increase of presumed ChIs firing and DA-LTD, through increased ACh release, was confirmed by the observation that DA-LTD was prevented under conditions of synaptic ACh depletion, due to chronic treatment of the slices with vesamicol and hemicholinium-3, antagonists of the vesicular ACh transporter and of choline reuptake, respectively, while the same treatment did not preclude the sustained ChIs firing potentiation by DHPG.

The crucial and delicate interplay between ACh and DA in the striatum has been thoroughly investigated in recent years, pointing to presynaptic nAChRs located onto the DAergic terminals as key substrates. In principle, their recruitment promotes DA release, as demonstrated by the nAChRs-induced release of DA in response to striatal ChIs stimulation (Threlfell et al., 2012; Brimblecombe et al., 2018). However, this faciliatory action through nAChRs appears to be subject to a subtle balance, whereby stronger stimulation of the same receptor may have an opposing action, causing DA release inhibition rather than facilitation, probably because of nAChRs desensitization. In fact, while the pharmacological block of nAChRs suppresses the evoked DAergic signal, nicotine or ACh esterase blockers inhibit, rather facilitate DA release (Zhou et al., 2001; Wang et al., 2014).

We thus propose that DA-LTD induced by DHPG is a secondary effect of the pronounced and sustained increase of released ACh, due to the parallel long-lasting potentiation of striatal ChIs firing.

Certainly, this hypothesis finds support in our present report only on pharmacology-based indirect observations, calling for more cautious conclusions. However, all these pharmacological results point towards this cellular mechanism, living little space for alternative interpretations. Moreover, in further support of this hypothesis, it is worth noticing that the increase in extracellular ACh by neostigmine was sufficient to strongly inhibit evoked DA release. However, it was rapidly restored after the drug washout. Conversely, the analogous effect of DHPG persisted at washout, at a time when ChIs maintain their increased firing rate, responsible for an enduring enhancement of the extracellular cholinergic tone.

A further element adding to the complexity in the local interplay between ACh and DA is represented by the role of mAChRs, expressed on the ChIs population and operating as a feedback mechanism on their membrane excitability (Weiner et al., 1990; Bernard et al., 1992; Bonsi et al., 2008; Zhao et al., 2016). The presence of an ongoing feedback between tonic ACh release and ChIs firing is evident also in our own data, showing that the block of ACh reuptake by neostigmine reduces the firing rate of striatal tonically active neurons in control conditions (Figure 5A). Thus, the increased firing of ChIs, triggered by mGluRs stimulation, is presumably occurring in spite of a concomitant increase in feedback inhibition mediated by postsynaptic mAChRs.

ChIs have long been known to express mGluR1 and 5, whose stimulation causes membrane depolarization (Pisani et al., 2001; Bonsi et al., 2005). The relatively longer period of mGluRs stimulation with DHPG (5 min) used in our protocol probably accounts for the sustained effect here reported and not previously observed. Analogous forms of intrinsic long-term plasticity, expressed as sustained changes in the postsynaptic membrane properties, have already been reported in other brain areas, often involving mGluR-mediated processes (Brager and Johnston, 2007; Brown et al., 2011; Hamlet and Lu, 2016; Tidball et al., 2017; Yu et al., 2018). The molecular and ionic mechanisms underlying the long-lasting hyperexcitability of striatal ChIs will be a subject of further investigation, focusing in particular on hyperpolarization-activated cyclic nucleotide-gated (HCN) and Ca^2+^ activated K^+^ conductances, based on their crucial role in striatal ChIs intrinsic firing (Choi et al., 2020).

Notably, LTD at hippocampal CA3-CA1 synapses, induced with a similar protocol of DHPG bath perfusion, undergoes a reversible recovery with MCPG and other mGluR antagonists, several minutes after DHPG washout (Lodge et al., 2013). In a similar manner, MCPG transiently reverted both DA-LTD and the sustained increase in ChIs firing by a previous DHPG perfusion. The precise mechanism underlying this effect has not been completely elucidated, although a long-lasting conformational change in mGluRs has been proposed so that they remain in a permanently active state, which can be deactivated by specific antagonists (Lodge et al., 2013).

### Functional Implications

Striatal dopaminergic, cholinergic, and glutamatergic transmission make an ensembled orchestra of multifaced reciprocal interactions, dynamically governed according to strength, as well as frequency codes of converging information, responsible for physiological and pathological behaviors (Zhang and Sulzer, 2004; Exley and Cragg, 2008; Cachope and Cheer, 2014; Sulzer et al., 2016). Within this picture, glutamate is known to be co-released by the DA terminals and act on ChIs through mGluRs in the dorsolateral striatum (Chuhma et al., 2004, 2018; Hnasko et al., 2010; Cai and Ford, 2018). Thus, our results suggest a dynamic crosstalk between the DAergic terminals and ChIs, whereby the former is capable to give rise to a sustained plastic increase of ChIs intrinsic excitability, through postsynaptic mGluRs stimulation, and consequently the latter responds by acting as a long-lasting feedback filter over the significance of the incoming DAergic signal. In addition, other glutamatergic inputs from the cortex and the thalamus might similarly participate in the regulation of this functional connection between ChIs and DAergic terminals.

On the other hand, this mGluR-mediated long-lasting increase of ChIs excitability also acts as a generalized braking factor limiting the DAergic input to the striatum. Accordingly, we found that when the group I mGluR agonist was bilaterally infused into the striatum, DHPG-treated mice displayed an anxiety-like behavior, that is typical of several neuropsychiatric disorders and is consistent with depletion of striatal DA (Magen and Chesselet, 2010; Lelos and Dunnett, 2011; Vieira et al., 2019), although a causal link between this behavioral response and an underlying cellular crosstalk between DAergic terminals and ChIs still needs a direct experimental demonstration.

This observation of emotional processes in response to DHPG injections, with no effect on motor activity—as evident in OF—agrees with previous results (Ztaou et al., 2018), while other authors report a significant increase in locomotion following striatal injection of mGluR agonists (Kearney et al., 1997). The difference in dose, species, brain area, protocols of drug application, and time of behavioral observation have to be taken into account. Noteworthily, DHPG effect on locomotor activity has been reported either attenuated, enhanced, or not modified, depending on the dose (Wiśniewski and Car, 2002).

Our behavioral results then add to the widely accepted notion of mGluR involvement in affective disorders, such as stress and anxiety. Specifically, modulation of mGluR5 and mGluR1 subtypes in the regulation of anxiety has received great attention pointing to mGluR5 and mGluR1 antagonists as novel anxiolytic-like drugs (Varty et al., 2005; Chen et al., 2011).

In conclusion, our data highlight the dorsal striatum as a key area linked to the development of anxiety-like behavior, proposing the hypothesis that this may occur through long-lasting plasticity processes affecting ChIs intrinsic excitability and DA release, triggered by mGluRs.

## Data Availability Statement

The raw data supporting the conclusions of this article will be made available by the authors, without undue reservation.

## Ethics Statement

The animal study was reviewed and approved by Animal Welfare Body at the Santa Lucia Foundation IRCCS.

## Author Contributions

MF and FR performed amperometric experiments. LM and NB performed multielectrode array recording experiments. SD’A and RV performed and designed behavioral experiments. NM and NB designed the experiments and wrote the manuscript. All authors reviewed the manuscript before submission. All authors contributed to the article and approved the submitted version.

## Conflict of Interest

The authors declare that the research was conducted in the absence of any commercial or financial relationships that could be construed as a potential conflict of interest.

## Publisher’s Note

All claims expressed in this article are solely those of the authors and do not necessarily represent those of their affiliated organizations, or those of the publisher, the editors and the reviewers. Any product that may be evaluated in this article, or claim that may be made by its manufacturer, is not guaranteed or endorsed by the publisher.
